# Lung volume assessments in normal and surfactant depleted lungs: agreement between bedside techniques and CT imaging

**DOI:** 10.1186/1471-2253-14-64

**Published:** 2014-08-05

**Authors:** Gergely Albu, Ferenc Petak, Tristan Zand, Magnus Hallbäck, Mats Wallin, Walid Habre

**Affiliations:** 1 Pathophysiological Experimental Platform, Department of Anaesthesiology, Pharmacology and Intensive Care, University of Geneva, 1 Rue Michel Servet, CH-1205 Geneva, Switzerland; 2 Department of Medical Physics and Informatics, University of Szeged, 9 Koranyi fasor, H-6720 Szeged, Hungary; 3 Paediatric Radiology Unit, Department of Radiology and Nuclear Medicine, University Hospitals of Geneva, 6 rue Willy Donzé, CH-1205 Geneva, Switzerland; 4 Maquet Critical Care AB, Röntgenvägen 2, 17154 Solna, Sweden; 5 Paediatric Anaesthesia Unit, Geneva Children’s Hospital, University Hospitals of Geneva, 6, Rue Willy Donzé, CH-1205 Geneva, Switzerland

**Keywords:** Effective lung volume, Capnodynamics, Gas exchange, Lung mechanics, Lung injury

## Abstract

**Background:**

Bedside assessment of lung volume in clinical practice is crucial to adapt ventilation strategy. We compared bedside measures of lung volume by helium multiple-breath washout technique (EELV_MBW,He_) and effective lung volume based on capnodynamics (ELV) to those assessed from spiral chest CT scans (EELV_CT_) under different PEEP levels in control and surfactant-depleted lungs.

**Methods:**

Lung volume was assessed in anaesthetized mechanically ventilated rabbits successively by measuring i) ELV by analyzing CO_2_ elimination traces during the application of periods of 5 consecutive alterations in inspiratory/expiratory ratio (1:2 to 1.5:1), ii) measuring EELV_MBW,He_ by using helium as a tracer gas, and iii) EELV_CT_ from CT scan images by computing the normalized lung density. All measurements were performed at PEEP of 0, 3 and 9 cmH_2_O in random order under control condition and following surfactant depletion by whole lung lavage.

**Results:**

Variables obtained with all techniques followed sensitively the lung volume changes with PEEP. Excellent correlation and close agreement was observed between EELV_MBW,He_ and EELV_CT_ (r = 0.93, p < 0.0001). ELV overestimated EELV_MBW,He_ and EELV_CT_ in normal lungs, whereas this difference was not evidenced following surfactant depletion. These findings resulted in somewhat diminished but still significant correlations between ELV and EELV_CT_ (r = 0.58, p < 0.001) or EELV_MBW,He_ (0.76, p < 0.001) and moderate agreements.

**Conclusions:**

Lung volume assessed with bedside techniques allow the monitoring of the changes in the lung aeration with PEEP both in normal lungs and in a model of acute lung injury. Under stable pulmonary haemodynamic condition, ELV allows continuous lung volume monitoring, whereas EELV_MBW,He_ offers a more accurate estimation, but intermittently.

## Background

The history of medicine has been marked by a spectacular evolution of mechanical ventilation and its inevitable benefit in providing life support both in anaesthesia and critical care management. Nevertheless, one of the main consequences of mechanical ventilation with positive intermittent pressure in the airways is the loss of static lung volumes subsequent to atelectasis, intra-alveolar fluid accumulation, interstitial edema formation, and surfactant damage. In order to prevent these deleterious effects on the lung, new concepts of mechanical ventilation target an open lung ventilation strategy involving protective ventilation modes with low tidal volumes, regular alveolar recruitments and maintenance of optimal positive end-expiratory pressure (PEEP). In order to optimize ventilation strategy, it is essential to include lung volume assessment as part of ventilation monitoring.

Assessment of lung volumes is widespread in the respiratory medicine; however, there are only few techniques that can be implemented at bed side to optimize the ventilation strategy. Static lung volume, such as the end-expiratory lung volume (EELV) in ventilated patients is assessed by analyzing the concentration changes of an inert gas during wash-in/wash-out maneuvers [1-4]. A recently developed alternative to EELV is the effective lung volume (ELV), which is based on a continuous breath-by-breath analysis of the carbon dioxide after alteration of the inspiratory pattern [5]. We showed that ELV detects lung derecruitment and recruitment [6] with the additional advantage of continuous monitoring of the ventilated patient.

Image processing to quantify lung volume from computed tomography CT scans has been largely used as a reference method in patients with acute lung injury [7-9]. However, it is technically demanding, time consuming and involves radiation exposure. CTs are therefore inappropriate for routine clinical practice. Bedside monitoring of EELV may provide similar insight into the lung volume available for gas exchange. Comparative results for these different available approaches for quantification of the aerated lung areas are not available.

We aimed to relate lung volume indices obtained from bedside techniques to those computed by chest CT image analyses. Furthermore, we aimed to establish the relationships between static lung volume outcome variables obtained from healthy lungs and in a model of acute lung injury by surfactant depletion.

## Methods

### Animal preparation

The experimental protocol was approved by the institutional ethics committee for experimental research of the University of Geneva and animal welfare committee (Office Véterinaire Cantonal de Genève registration number 1051/3609/1, Geneva, Switzerland). Eight adult New Zealand white rabbits (weighing 2.4-3.1 kg) were anaesthetized by an intramuscular injection of xylazine (5 mg/kg), followed by an iv injection of midazolam (1 mg/kg) and pentobarbital sodium (30 mg/kg) via an ear vein. Following tracheotomy, an endotracheal tube (4 mm i.d., Portex®, Smiths Medical, Kent, UK) was inserted into the distal trachea. Mechanical ventilation was started in volume controlled mode (Servo-i Maquet Critical Care, Solna Sweden equipped with an additional software) with a fixed respiration rate of 40 breaths/min and inspired oxygen fraction (FiO_2_) of 0.5. Tidal volume was set to 7-8 ml/kg in order to target an end-tidal CO_2_ (E_TCO2_) of 5.5-6%. Maintenance of anesthesia was assured by a continuous iv infusion of midazolam (1 mg/kg/h) and fentanyl (100 μg/kg/h) via the ear vein. Muscle relaxation was achieved by atracurium (0.5-1.0 mg/kg/h) after ensuring adequate anesthesia and analgesia level. Airway pressure, heart rate and rectal temperature were displayed and stored on a computer at a sampling rate of 50 Hz via an analogue/digital interface converter (Biopac, Santa Barbara, CA, USA).

### Measurement of EELV by multiple breath washout technique

A technique using multiple-breath wash-in/wash-out maneuvers with helium (He) as tracer gas was used to measure EELV_MBW,He_, as detailed previously [1,10]. Briefly, an ultrasonic flow meter (Spiroson Scientific; ECO Medics AG, Dürnten, Switzerland) was instrumented between the endotracheal tube connector and the ventilator circuit to assess changes in molar mass of the respiratory gas. A recording at steady-state end-inspiratory concentration of He at 4-5% was established. by washing-in the inert gas into the ventilatory circuit. The He tracer gas flow was interrupted, and the washout of the tracer gas was recorded. EELV_MBW,He_ was calculated from the changes in the He concentration during the wash-out phase using the software, (Spiroware, V1.4.3, ECO Medics AG, Dürnten, Switzerland) as:

$$\mathrm{EEL}V_{\mathrm{MBW},\mathrm{He}}=\frac{\mathrm{net}\;\mathrm{volume}\;\mathrm{of}\;\mathrm{inert}\;\mathrm{gas}\;\mathrm{exhaled}}{C_{\mathrm{start}}-C_{\mathrm{end}}},$$

where C_start_ and C_end_ are the concentrations of He at end-tidal volume at the start and at the end of the He wash-out recording, respectively. The multiple breath washout technique also allows the assessment of the lung clearance index (LCI) as an indicator of ventilation heterogeneity. LCI was calculated as the number of lung volume turnovers required to diminish the He concentration to 1/40th of the starting value [11]. The instrumental dead space (3.8 ml) was subtracted from the reported EELV_MBW,He_ values.

### Assessment of ELV

Ventilation airflow and changes in CO_2_ concentration were recorded by ordinary Y-piece flow sensor and a main stream capnometer of the Servo-i ventilator. Flow and CO_2_ signals were digitized by a computer via an RS232 port. Analyses of the flow and CO_2_ traces were performed by using a special purpose software application written in Matlab™ (Mathworks, Natick, Massachusetts, USA).

Breath-by-breath analysis of the CO_2_ changes after alteration of the inspiratory pattern was used to determine the effective lung volume that takes part in gas exchange, as detailed previously [6]. Briefly, the inspiratory pause was varied to introduce 5 consecutive alterations in inspiratory/expiratory ratio (1:2 to 1.5:1) to the normal mechanical ventilation. Resulting from this modified breathing pattern, a variation in end-tidal carbon dioxide (E_TCO2_) of about 0.5-1.0 kPa develops. The differential Fick’s method can be used continuously without attaining a new second steady-state condition by measuring and calculating the dynamic transient changes in E_TCO2_ and CO_2_ elimination rate [12].

An equation describing the mole balance in the lungs was used to calculate ELV:

$$\mathrm{ELV}\cdot \left(F_{A}CO_{2}^{n}-F_{A}CO_{2}^{n‒1}\right)=Q_{c}\cdot \Delta t^{n}\cdot \left(C_{v}CO_{2}-C_{c}CO_{2}^{n}\right)-VT_{\mathrm{CO}2^{n}},$$

where ELV is the effective lung volume containing CO_2_ at end of expiration, n is the current and n-1 is the previous breath, F_A_CO_2_ is the alveolar CO_2_ fraction at end of expiration (approximated by E_TCO2_), Q_c_ is the effective pulmonary blood flow, C_v_CO_2_ is the venous CO_2_ content [L_gas_/L_blood_], C_c_CO_2_^n^ is the lung capillary CO_2_ content (calculated from E_TCO2_), VT_CO2_^n^ is the volume of CO_2_ eliminated by the current (n^th^) breath and Δt^n^ is the time for the current breath cycle time. The left side of the equation yields the difference in end-tidal CO_2_ content in the lung between two breaths. The first term on the right side describes the circulatory supply of CO_2_ in the alveolar compartment between two breaths. The CO_2_ content in the lung capillary blood, C_c_CO_2_, is calculated from the alveolar CO_2_ fraction using the dissociation curve suggested by Capek et al. [13]. The second term on the right side is the amount of carbon dioxide eliminated from the lungs by the n^th^ tidal volume.

To determine the unknown variables, a new equation is created for each of the 10 breaths in the sequence. Thus, 10 breaths create 10 equations with three unknown variables. The equation system can be solved by optimizing the fit between observed F_A_CO_2_ data and calculated F_A_CO_2_ according to the balance equation above. Thus, it is possible to determine ELV, which is the gas volume in the lung at end-expiration including the airway volume up to the site of the CO_2_-sensor.

### Assessment of EELV by CT image processing

The CT scans were taken in the supine position using a high resolution CT scanner (GE LightSpeed VCT 64, Waukesha, Wisconsin, USA). The scanning time was fixed on 2.5 s, helical full in 0.4 s. The field of view was 20 cm and the CT-scanning was set at energy of 100 kV, and an automatic electrical current between 10 to 200 mA, with a signal-to-noise ratio of 10. The image thickness was 0.625 cm with identical image interval. A standard high resolution reconstruction algorithm for the lung was employed. During the scanning periods, the mechanical ventilation was suspended at end-expiration. No contrast medium was injected.

The pulmonary volumes were calculated on the CT scan by manual surface delineation of pulmonary tissue on the transverse slices multiplied by slice thickness. As there are many interstitial structures and some focal infiltrates and hypoventilation beyond the CT resolution, the volume was compensated to better approximate actual air content by pondering the total volume by a factor based on the mean Hounsfield Unit measurement of the same pulmonary area. The pondering factor was calculated based on the measurement of air outside the actual body (approximately -1000HU), and that all other tissues density can be well evaluated by a ROI on the hilum (approximately 0HU), and the linear nature of HU density characteristics.

### Experimental protocol

**Figure 1:**
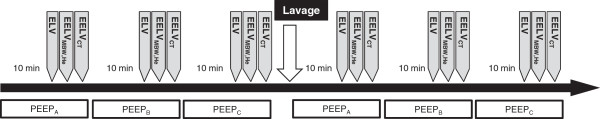
Scheme of the experimental protocol.

After the surgical preparation, the rabbits were ventilated with PEEP of 3 cmH_2_O and a 10-15 min period was allowed for the animals to reach a steady-state condition in the systemic hemodynamic and ventilation parameters. The “pressure history” was then standardized by superimposing inspiratory cycles to reach a peak pressure of 30 cmH_2_O. The first PEEP level was then set (either 0, 3 or 9 cmH_2_O) and continuous measurement of ELV with the Servo-i ventilator was then performed for 10 minutes before recording its steady-state value (Figure 1). Two minutes later, two multiple breath wash-in/wash-out maneuvers with He were performed to assess EELV_MBW,He_. These lung volume measurements were followed by the chest CT imaging. The same sequence of measurements including the standardization of the “pressure history” was then repeated twice while setting the PEEP to the other two levels in random order. After completing the lung volume recordings in the healthy lungs, whole lung lavage was performed by instilling warm 0.9% saline (15 ml/kg at 37°C) into the endotracheal cannula. The instilled fluid was then withdrawn by gentle manual suctioning. This surfactant depletion procedure was repeated twice with the animal being reconnected and ventilated between the manoeuvres. After lavage, FiO_2_ was increased to 80%, the standardisation manoeuvre was performed as detailed above and the lung volume measurements were repeated while PEEP levels of 0, 3 and 9 cmH_2_O were maintained in random order.

### Statistical analyses

The scatters in the variables were expressed by the SE values. Normality of the data was checked with the Kolgomorov-Smirnov test with Lilliefors correction. Two-way repeated measures analysis of variance (ANOVA) was used with the PEEP level as the within subject factor and the lung volume assessment method as the between subject factor to establish the differences between the assessment methods and PEEP. Pairwise comparisons were performed on estimated marginal means by taking into account the presence or absence of interaction; the p-values were corrected by the Holm-Sidak method. Correlation analyses were performed by using Pearson statistical tests. A modified polar plot representation of the Bland-Altman analysis [14] that allows more elaborate trend assessment was performed to determine the agreement between different lung volume indices [15,16]. In this presentation, the angle between the radial vector and the horizontal axis represents the magnitude of difference between the corresponding variables, while the distance from the center represents the mean of the two corresponding variables. Statistical tests were carried out with the significance level set at p < 0.05. SigmaPlot software package (version 11, Chicago, IL, USA) was used in the analyses.

## Results

**Figure 2:**
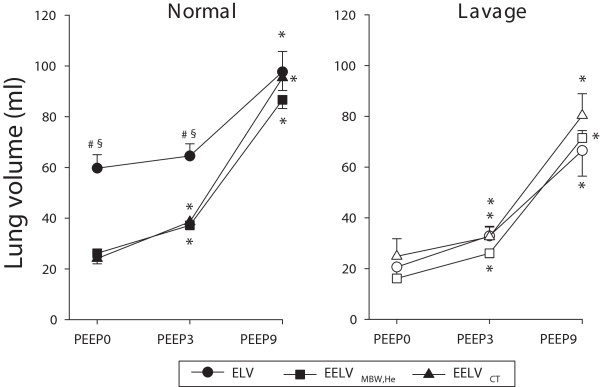
**Lung volume variables obtained at different PEEP levels in normal (closed symbols) and surfactant depleted lungs (open symbols).** Circles denote the mean values for effective lung volume (ELV) obtained by capnodynamics; squares represent mean values obtained by helium multiple breath washout technique (EELV_MBW,He_); and triangles represent mean values for lung volume computed from chest CT-scans (EELV_CT_). *: p < 0.05 vs. PEEP0, #: p < 0.05 vs. EELV_MBW,He_ within a PEEP, §: p < 0.05 vs. EELV_CT_ within a PEEP.

**Figure 3:**
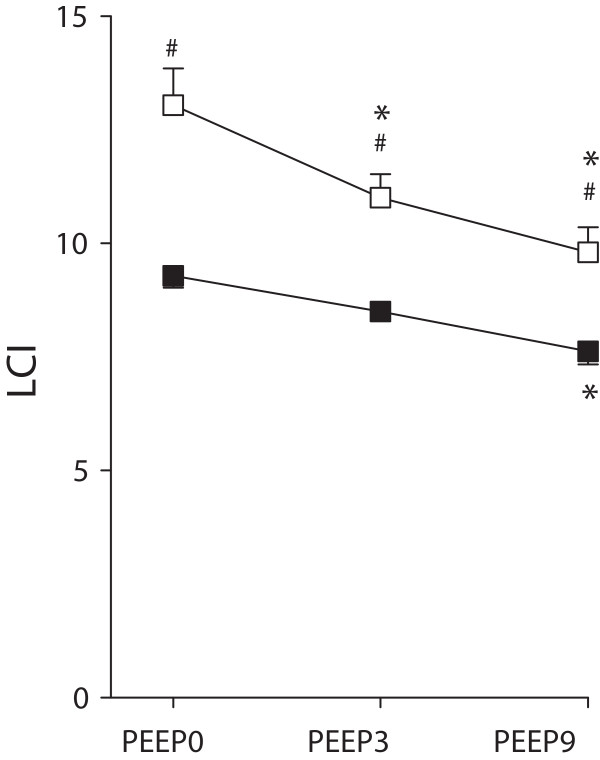
**Lung clearance index (LCI) obtained by helium multiple breath washout technique at different PEEP levels in normal (closed symbols) and surfactant depleted lungs (open symbols).** *: p < 0.05 vs. PEEP0, #: p < 0.05 normal vs. surfactant depletion within a PEEP.

Lung volume indices obtained at different PEEP levels in healthy and surfactant-depleted lungs are demonstrated in Figure 2. As expected, increasing PEEP led to elevations in all end expiratory lung volume variables before (p < 0.001) and after whole lung lavage (p < 0.001). In the healthy lungs, no statistically significant differences were evident between EELV_MBW,He_ and EELV_CT_ at any PEEP levels. However, ELV was significantly greater than either EELV variable in healthy lung at low PEEP levels (p < 0.001) while this difference was not obvious during the maintenance of a PEEP of 9 cmH_2_O. No statistically significant difference was detectable between the lung volume variables after lung lavage at any PEEP level.Figure 3 depicts changes in the LCI with PEEP under baseline conditions and after lung lavage. Surfactant depletion elevated LCI at all PEEP levels (p < 0.001). Increasing PEEP resulted in significant decreases in the LCI (p < 0.001); these reductions were more pronounced for the lungs after lavage.

**Figure 4:**
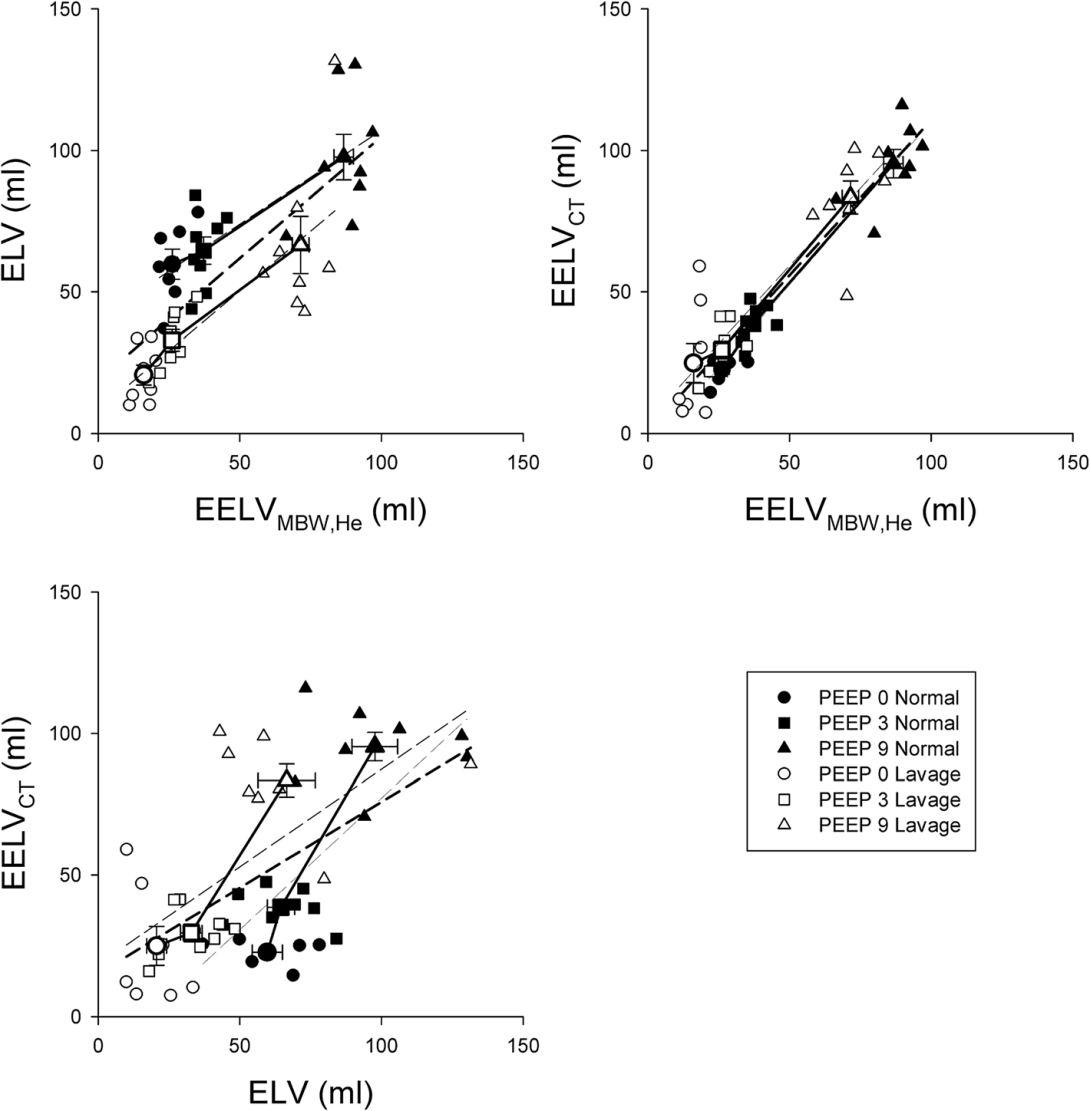
**Relationships between pairs of lung volume indices obtained by capnodynamics (ELV), by helium multiple breath washout technique (EELV**_**MBW,He**_**) and by chest CT-scans (EELV**_**CT**_**).** Closed symbols represent lung volume measurements in normal lungs while open symbols denote the values obtained after lavage. Individual values (small symbols) and group mean values (larger symbols) are reported. Dashed lines represent linear regressions with thin lines denoting each condition separately and thick line corresponds to pooled data.

The relationship between the lung volume indices obtained by the three different measurements techniques are demonstrated on Figure 4. There were statistically significant correlations between each pair of variables (p < 0.001) with strongest associations between EELV_MBW,He_ and EELV_CT_ (r = 0.93, p < 0.0001). The correlations between the ELV and the other two lung volume indices (EELV_CT_ and EELV_MBW,He_) were somewhat weaker (r = 0.58 and r = 0.76), but still highly significant (p < 0.0001).

**Figure 5:**
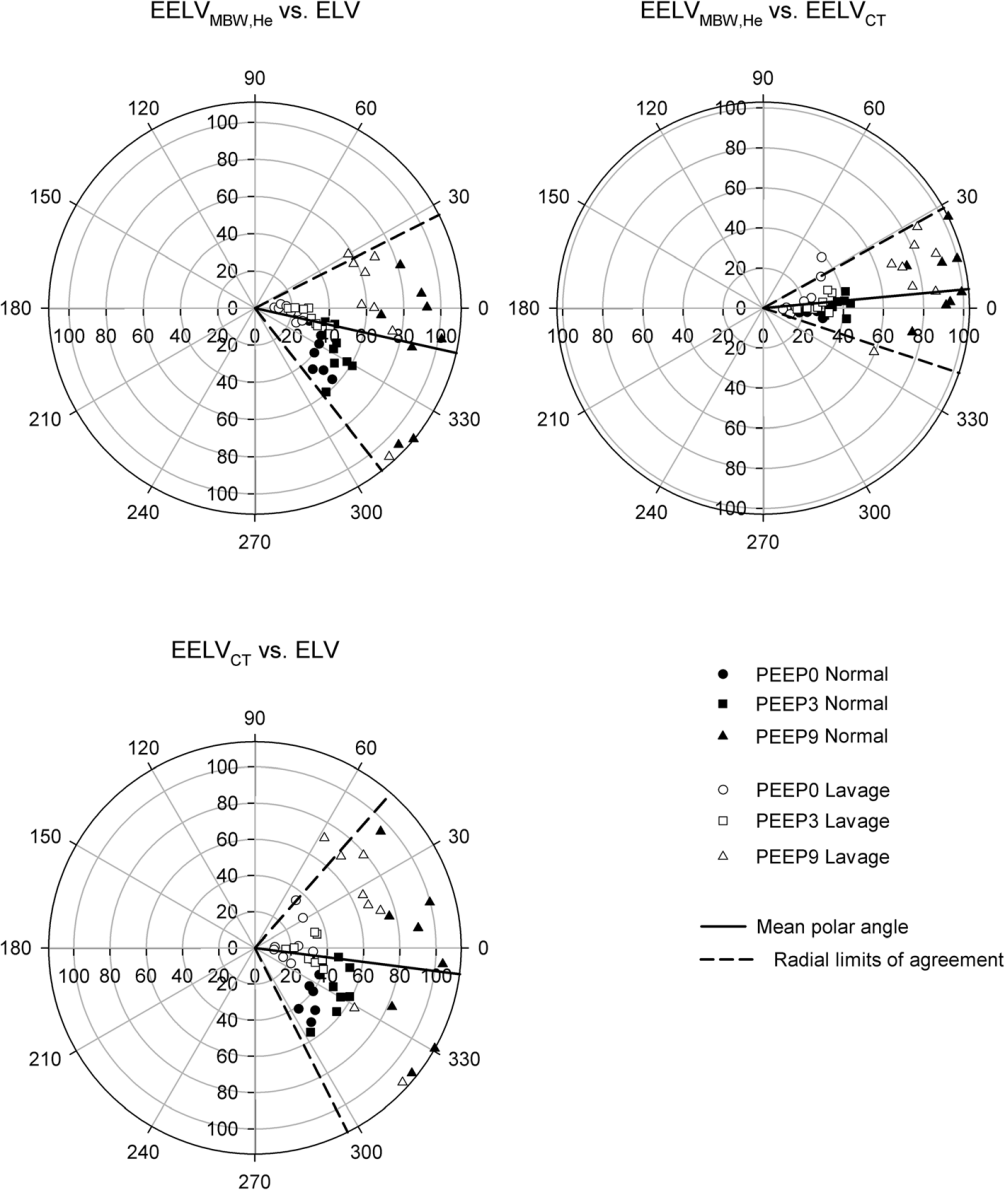
**Polar plots demonstrating the degree and the trend of agreements between the lung volume indices.** Closed symbols on the left panels represent lung volume measurements in normal lungs while open symbols on the right panels denote the values obtained after lavage. The angle between the radial vector and the horizontal axis represents the magnitude of difference between the corresponding variables (in ml), while the distance from the center represents the mean of the two corresponding variables (in ml). Solid lines indicate the mean polar angles, while thick dashed lines denote the radial limits of agreements.

Figure 5 summarizes the degrees of agreements between each pair of lung volume variables measured on polar plots. The mean value of the variable pairs are plotted as a distance from the centre of the polar plot, while the angle between the radial vector and the horizontal axis represents the differences between each pair of variables. Excellent agreement was found between EELV_MBW,He_ and EELV_CT_ in the normal lungs with mean polar angle of 2.4 degrees and radial limits of agreement of -15.5 and 20.3 degrees (based on the 95% confidence interval limits). This close agreement remained after lavage with mean polar angle of 8.0 degrees and agreement limits of -19.6 and 35.6 degrees. There was a weaker agreement between the ELV and the other two lung volume variables, with the mean polar angles of -23.5 (EELV_MBW,He_) and -21.1 (EELV_CT_) degrees and radial limits of agreement of -58.9 and 11.8 (EELV_MBW,He_), and -69.3 and 27.1 degrees (EELV_CT_), respectively, under baseline condition. Surfactant depletion improved these agreements with mean polar angles of -2.1 (EELV_MBW,He_) and 5.8 (EELV_CT_) degrees and radial limits of agreement of -34.1 and 29.8 (EELV_MBW,He_), and -45.6 and 57.4 degrees (EELV_CT_), respectively.

## Discussion

The results of the present study demonstrate that bedside assessment of lung volume offers a meaningful alternative to those obtained by CT scan imaging. Both bedside lung volume assessments were able to follow the increased lung aeration with elevating PEEP and the development of airway closures following surfactant depletion. This study also evidenced an excellent value of the lung volume measurement performed by inert gas multiple breath washout technique both in normal lungs and in a model of acute lung injury. Conversely, effective lung volume available for gas exchange assessed from the analyses of the expired CO_2_ exhibited greater scatters in the agreements with the other two indices.

We adopted the technique of surfactant depletion by performing whole lung lavage as a model of acute lung injury. The pathogenesis of this severe pulmonary disorder is far more complex then the loss of surfactant function. Nonetheless, the present model mimics lung collapsibility and the loss or regional lung ventilation with increased heterogeneity, particularly at low PEEP levels [17]. Atelectasis development was evidenced from the marked and significant decreases in EELV_MBW,He_ (-38 ± 5.3%, p < 0.005) and ELV (-65 ± 4.2%, p < 0.001) at PEEP 0. The lavage-induced changes in EELV_CT_ were less obvious, since lung volume variable includes all aerated structures including those with trapped air [17]. To avoid time effects in our findings, the order of PEEP was randomized within a set of recordings. However, the three different lung volume readings were always performed in the same sequence. None of the measurements techniques required altering the ventilation pressures beyond the normal range. The only change in the ventilation pattern was necessary to record ELV, but the short inspiratory pause did not affect the peak or mean airway pressures markedly and thus, it was unlikely to bias our findings.

An important finding of the present study is that while ELV follows the expected trend with increasing PEEP, it systematically overestimates the EELV at low PEEP levels in the healthy lungs (Figure 2). To provide a plausible explanation for this apparent discrepancy, the fundamental differences between ELV and EELV estimates warrant consideration. ELV reported in the present study reflects all compartments contributing to the CO_2_ content in the lung including the alveoli, lung tissue and lung capillary blood. While the individual contribution of these compartments is not straightforward to assess in the present study, previous investigations established a correction factor of 55% at PEEP 0 in healthy lungs to adjust for both blood and lung tissue CO_2_ content [18]. Indeed, implementation of this correction factor at PEEP 0 reveals excellent agreements between the corrected ELV (26.9 ± 2.4 ml), the EELV_MBW,He_ (26.2 ± 1.8 ml) and the EELV_CT_ data (24.1 ± 2.1 ml). Nevertheless, a systematic application of such a correction factor during the maintenance of different PEEP levels and in injured lungs would be erroneous, since both PEEP and surfactant depletion affect markedly the capillary blood in the alveolar wall and the parenchymal contribution to the total CO_2_[19]. A PEEP-dependent correction factor taking into account the capillary blood volume and the lung tissue contribution in healthy and injured lungs may be a subject for further investigations.

When different lung volume indices and their changes are related, an important feature of their ability to access trapped (non-ventilated) lung regions should be taken into account. Since EELV and ELV are assessed by analyzing the dynamics of the expired gas, these measures provide information only about the size of the ventilated lung regions. Conversely, quantification of lung volume based on chest CT image analyses includes all low attenuation areas and thus, it cannot distinguish between ventilated or trapped aerated regions. This difference is unlikely to affect the lung volume estimates in a normal lungs, however may gain importance after lung lavage where the airway closures may lead to trapped air in the lung periphery. Using a similar experimental model where the regional lung ventilation was assessed by synchrotron imaging, we recently demonstrated that air trapping may be encountered at PEEP level of 3 cmH_2_O [17]. Considering that the maximum amount of such lung regions with trapped air does not exceed 10%, the impact of this phenomenon on our results is minor. Accordingly, we observed no significant difference between the results of the three measurement techniques after lung lavage, despite a trend for a greater EELV_CT_ than the other two lung volume indices that may be explained by this factor (Figure 2, right).

To our knowledge, this is the first study relating lung volume variables obtained by a continuous assessment (ELV) to wash-in/wash-out and imaging techniques in ventilated subjects. A remarkable finding of the present study is the excellent concordance between the lung volume variables obtained by inert gas washout and chest CT imaging. This was manifested both in the excellent correlations between EELV_MBW,He_ and EELV_CT_ (Figure 4) and the good agreement (Figure 5). The importance of this finding stems from the fact that multiple breath washout technique by using ultrasonic flow meter has been applied commonly in experimental [6,10,17,20] and clinical studies [2,21-26]. However, the relationship of EELV_MBW,He_ obtained by this approach was not related to that obtained by using a reference method in patients with acute lung injury based on CT imaging [7], and the validity of this variable has not been evaluated. Therefore, the results of the present study provides additional evidence on the validity of this ultrasonic tool for monitoring lung volume changes since it provides essentially identical information than the more cumbersome CT-imaging. Moreover, the application of the multiple breath wash-out technique gives quantitative information about the magnitude of ventilation heterogeneities, which may be another important variable to guide optimal lung ventilation without the need for the costly imaging involving radiation exposure [27,28].

The advantage of ELV as opposed to the other two lung volume indices is that it allows real-time monitoring of the ventilated lung area available for gas exchange. The results of the present study agree with our previous findings demonstrating that ELV systematically overestimates lung volume measured by inert gas washout in normal lungs at low PEEP levels. This discrepancy disappeared at high PEEP level in normal lungs and was no longer detectable at any PEEP after lung lavage. This divergent relationship resulted in a poorer but significant correlation and worsened the agreement between ELV and EELV_MBW,He_ in normal and surfactant depleted lungs (Figures 4 and 5). The novelty of the present study in this regard is the demonstration of a similar relationship of ELV with changes in lung volume computed from chest CT scans. The systematic overestimation of static lung volumes by ELV is most plausibly explained by the contribution of lung capillary blood volume to ELV. Since ELV estimation is based on the analyses of the CO_2_ as a tracer gas, it comprises the total CO_2_ content of the lung, which is the sum of the CO_2_ in the blood and in the alveolar space [6]. Thus, a larger lung volume is expected from the soluble CO_2_ estimates as compared to a method based on non-soluble tracer gas, such as Helium, which solely measures the alveolar gas volume. Applying an elevated PEEP decreases the capillary blood volume in the pulmonary circulation [19], and this results in a better agreement between ELV and other lung volume indices when high positive pressure is applied in the airways. Furthermore, airway closures induced by surfactant depletion compromises CO_2_ diffusion into the airspaces, which may explain the improved correlation and closer agreement between ELV and the other lung volume variables in the present of diminished surfactant function. These considerations highlight the potential susceptibility of ELV to alterations in ventilation/perfusion mismatch.

## Conclusions

Lung volume assessment is required to monitor the development of atelectasis and alveolar recruitment and to guide optimal mechanical ventilation. However, lung volume indices that can be obtained bedside have not been related to reference values measured by chest CT scans. Therefore, this study provides meaningful information about the usefulness of bedside monitoring of lung volume to follow its changes with PEEP in normal lungs and in a model of acute lung injury. While lung volume variables obtained by multiple breath washout, CO_2_ analyses and chest CT scans indicate lung expansion with increasing PEEP and alveolar derecruitment with lavage, their relationships are variable. Close correlation and excellent agreements were found between lung volumes obtained by He washout and chest CT analyses. However, the association between the effective lung volume achieved by analyzing the dynamics of expiratory CO_2_ concentration and the other variables proved to be less clear-cut. This may be attributed to methodological differences with particular involvement of the capillary blood volume in the ELV estimates. In conclusion, both bedside techniques offer a valuable tool to follow lung volume changes without the need for cumbersome intervention. However, lung volume monitored by the CO_2_ tracing provides continuous assessments that are subjected to pulmonary hemodynamical changes, while the EELV_MBW,He_ provides more specific measurements but at a cost of intermittent recordings.

## Abbreviations

C_c_CO_2_: Lung capillary CO_2_ content; CO_2_: Carbon dioxide; CT: Computed tomography; C_v_CO_2_: The venous CO_2_ content; ELV: Effective lung volume; EELV_MBW,He_: End-expiratory lung volume assessed by using multiple-breath washout technique; EELV_CT_: End-expiratory lung volume assessed by using chest CT imaging; E_TCO2_: End-tidal CO_2_ concentration; F_A_CO_2_: The alveolar CO_2_ fraction at end of expiration; FiO_2_: Inspired oxygen fraction; He: Helium; HU: Hounsfield Unit; LCI: Lung clearance index; PEEP: Positive end-expiratory pressure; Q_c_: Effective pulmonary blood flow; VT_CO2_: Volume of CO_2_ eliminated by one breath; Δt: Time for the breath cycle.

## Competing interests

The laboratory where the experiments were performed received a fund from Maquet Critical Care AB, Solna, Sweden to partially cover the material costs. Mats Wallin, Magnus Hallbäck work at the Research Development Department of the Maquet, Solna, Sweden and they established the theoretical bases of the ELV measurement technique based on capnodynamics. Walid Habre received a research grant from Maquet Solna, Sweden for acting as consultant in the respiratory developments.

## Authors’ contributions

GA and TZ carried out the experiments and the preliminary data analyses. FP coordinated the various experimental approaches, and contributed to their design and to the manuscript preparation. MW and MH participated in the design of the study and in the interpretation of ELV measurements. WH conducted the design of the study and had a major role in drafting the manuscript. All authors have read and approved the final manuscript.

## Pre-publication history

The pre-publication history for this paper can be accessed here:

http://www.biomedcentral.com/1471-2253/14/64/prepub
